# CD161, a promising Immune Checkpoint, correlates with Patient Prognosis: A Pan-cancer Analysis

**DOI:** 10.7150/jca.63236

**Published:** 2021-09-09

**Authors:** Wenrui Ye, Cong Luo, Chenglong Li, Zhixiong Liu, Fangkun Liu

**Affiliations:** 1 Department of Neurosurgery, Xiangya Hospital, Central South University, 87 Xiangya Road, Changsha, Hunan, 410008, China.; 2 Department of Urology, Xiangya Hospital, Central South University, 87 Xiangya Road, Changsha, Hunan, 410008, China.; 3 National Clinical Research Center for Geriatric Disorders, Xiangya Hospital, Central South University, 87 Xiangya Road, Changsha, Hunan, 410008, China.

**Keywords:** CD161, biomarker, cancer, bioinformatics, prognosis, immune infiltration

## Abstract

**Background:** CD161 is a promising immune checkpoint mainly expressed on natural killer (NK) cells and is essential for immunoregulatory functions. However, it remains obscure how CD161 correlates with immune infiltration and patient prognosis in pan-cancer.

**Methods:** We employed HPA, TCGA, GTEx, TIMER2.0, and GEPIA2 databases as well as R language to analyze and visualize CD161 in cancers. Our twenty-four glioma samples were sequenced for validation.

**Results:** Overall, CD161 was differentially expressed between most paired cancer and normal controls. Higher CD161 expression was associated with poorer overall survival (OS) in the TCGA LGG (HR = 2.18, 95%CI = 1.79-2.66, *P* < 0.001) and UVM (HR = 1.32, 95%CI = 1.05-1.65, *P* = 0.016) cohorts. In these two cancer types, CD161 was significantly correlated with expression levels of recognized immune checkpoints and the abundance of markers of specific immune subsets, including CD8+ T cells, dendric cells (DCs), M2 macrophages, and exhausted T cells (Texs). In addition, CD161 was involved in several immune pathways in LGG and UVM, highlighting its role in regulating immune processes in the context of oncology.

**Conclusions:** CD161 is a potential prognostic biomarker and immunotherapy target in human cancers, especially brain lower grade gliomas.

## Introduction

Cancer has gradually become the top killer threatening human life over the past few decades 1. Fortunately, with the deeper understanding of the intrinsic mechanisms underlying oncogenesis, researchers have launched more options for combating cancer 2. Flourishing efforts to target co-inhibitory or immune checkpoint receptors [For example, cytotoxic T lymphocyte associated protein 4 (CTLA-4) and programmed death-1 (PD-1)] responsible for an immunosuppressive phenotype, have shown great success in the treatment across various cancer types 3, 4. Despite the great success of immune checkpoint blockade (ICB), a significant number of patients do not respond to currently available immunotherapies or acquire resistance after a certain treatment duration 5. Thus, this conundrum has attracted attention for exploring novel immune checkpoints that can be safely targeted with high anti-tumor efficacy in the hope of achieving high response rates and better therapeutic outcomes.

CD161, encoded by killer cell lectin-like receptor subfamily B member 1 (*KLRB1*), is a C-type lectin-like receptor expressed on human natural killer NK cells 6 and various T lymphocyte subsets 7. CD161 may act as a lectin, binding to terminal carbohydrate gal alpha (1,3) gal epitopes as well as N-acetyllactosamine epitopes. As for the role in immunity, activation of CD161 resulted in significantly enhanced anti-CD3-induced T cell proliferation. In addition, it also binds to CLEC2D/LLT1 as a ligand (similar to PD-1/PD-L1 axis) and inhibits NK cell-mediated cytotoxicity as well as interferon-γ secretion in target cells.

Leveraging single-cell RNA sequencing (RNA-seq) analysis of tumor-infiltrating T cells in glioma patients, a recent study identified a T cell population co-expressing a cytotoxicity program and natural killer cell receptors. Specifically, genetic inactivation of *KLRB1* or antibody-mediated CD161 blockade enhances T cell-mediated killing of glioma cells *in vitro* and their anti-tumor function *in vivo* 8, identifying CD161-CLEC2D pathway as a potential target for immunotherapy. Furthermore, *KLRB1* was also expressed by a subset of tumor-infiltrating CD4+ and CD8+ T cells in other tumor types including melanoma, non-small cell lung cancer (NSCLC), hepatocellular carcinoma, and colorectal cancer indicated by several published scRNA-seq datasets 9-12.

However, studies on CD161 in other tumor types have rarely been reported yet, which makes it inconclusive whether CD161 functions as an immune checkpoint. More comprehensive analysis of CD161 profile in human cancer is warranted to understand the intrinsic role of CD161 in tumor immunity. We herein conducted a pan-cancer analysis to illustrate the profile of CD161, including expression, mutation status, correlation with signatures of interest, and contribution to patient survival. In this study, all data was elicited from widely applied open databases, and all these analyses were performed based on webtools and R language, version 4.0.2.

## Materials and Methods

### Data Source and Processing

The Cancer Genome Atlas (TCGA) is a landmark cancer genomics program, which has molecularly characterized over 20,000 primary cancer and matched normal samples spanning thirty-three cancer types until April, 2021. We collected CD161 data from various cancer samples in the TCGA database 13. Fragments per kilobase million (FPKM) values were transformed into transcripts per kilobase million (TPM) values, and were further log transformed for better comparisons between samples. The genotype-tissue expression (GTEx) database provides publicly available gene expression data from fifty-four normal tissue sites across nearly 1000 people 14. Normal samples from both TCGA and GTEx databases were integrated for comparisons between cancer and normal tissues.

### CD161 Expression Profiles

The Human Protein Atlas (HPA) is a program for mapping human proteins in cells, tissues and organs using integration of various omics technologies 15, 16. We obtained the immunohistochemistry results of CD161 proteins in certain tissues (including tumor and normal tissues) in the tissue atlas and pathology atlas in HPA database.

### Survival Analysis

We used the TCGA data and performed survival analysis, computed the log-rank P value and hazard ratio (HR) with 95% confidence interval (95%CI). The results were displayed as forest plots (using “forestplot” package in R) and survival curves (using “survival” package in R).

### Correlation Analysis

The correlations between CD161 and immune checkpoints (including but not limited to PDCD1, CD28 and CTLA4) were calculated 17. In addition, we examined the relationship between CD161 expression and genomic alteration signatures, including tumor mutation burden (TMB), microsatellite instability (MSI), mismatch repair (MMR) genes as well as DNA methyltransferases. The mismatch repair mechanism plays a key role in the identification and repair of mismatched bases during DNA replication and genetic recombination 18. MMR deficiency and subsequent MSI, a hypermutator phenotype subsequent to frequent polymorphism in short repetitive sequences and single nucleotide substitution 19, lead to accumulated TMB 20. Those genetic indicators besides DNA methyltransferases are closely linked to tumorigenesis and considered as independent predictors of immunotherapy efficacy 21, 22.

### Immune Infiltration and Enrichment Analysis

Tumor purity was estimated using “estimate” package in R. Stromal and Immune score represented the abundance of stromal and immune components, respectively. ESTIMATE score, the sum of stromal and immune scores, represents tumor purity to some extent. Tumor IMmune Estimation Resource 2.0 (TIMER2.0) web server is a comprehensive resource for systematic analysis of immune infiltrates across diverse cancer types 23, 24. We assessed the correlations between CD161 and six infiltrating lymphocytes, as well as the markers for immune cell subsets including CD8+ T cells, total T cells, B cells, monocytes, tumor-associated macrophages (TAMs), M1 and M2 macrophages, neutrophils, NK cells, DCs, Th1 cells, type 2 helper T cell (Th2), Tfh cells, type 17 helper T cell (Th17), Tregs, exhausted T cells, and follicular dendritic cells (FDC) 25, 26. Those correlations were re-analyzed using Gene Expression Profiling Interactive Analysis 2 (GEPIA2) 27, 28.

### RNA Sequencing of Glioma Samples

We included twenty-four glioma samples diagnosed at the Department of Neurosurgery, Xiangya Hospital from June 2018 to July 2021. Patients with the presence of other cancers or serious underlying diseases were excluded. Their clinical information was summarized in Table S1.

In brief, the fresh glioma samples were collected and then immediately stored in liquid nitrogen. Total RNA was extracted from the tissues using TRIzol (Invitrogen, Carlsbad, CA, USA) according to the instructions. Subsequently, NanoDrop and Agilent 2100 bioanalyzer (Thermo Fisher Scientific, MA, USA) were used to quantify total RNA, which was purified and fragmented into small pieces. Then, first- and second-strand cDNA were synthesized. The cDNA fragments were further amplified by polymerase chain reaction (PCR) after incubating with A-tailing mix and RNA Adapter Index for end repair. The qualified double-stranded PCR products were then used to construct the final library (single-stranded circular DNA). Eventually, the 24 qualified glioma samples were further sequenced on a BGISEQ-500 platform (BGI-Shenzhen, China). The gene expression levels were calculated using RSEM (v1.2.12).

### Statistical Analysis

Student's t-test and analysis of variance (ANOVA) test were used for comparisons between two groups and for comparisons of more than two groups, respectively. Spearman's correlation analysis was used to measure the degree of correlation between certain variables, and the following R/rho values were used to judge the correlations: 0-0.19, 'very weak'; 0.20-0.39, 'weak'; 0.40-0.59, 'moderate'; 0.60-0.79, 'strong'; 0.80-1.00, 'very strong'. In most analysis, *P* < 0.05 was the significance threshold.

## Results

### *KLRB1*/CD161 Expression Profiles in Human Cancers

**Figure 1:**
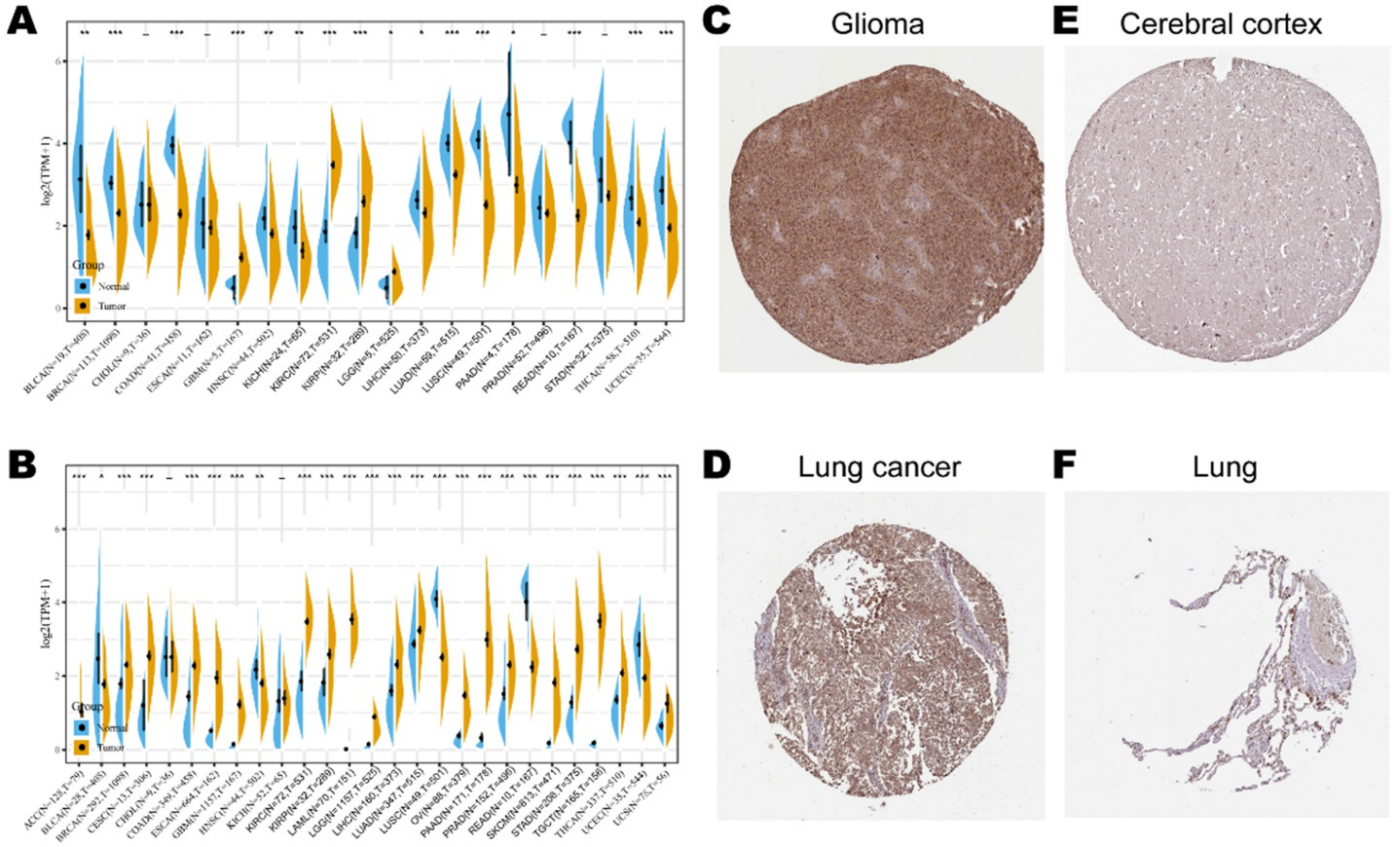
*KLRB1*/CD161 expression levels in different cancer types. (**A**) The expression level of *KLRB1* mRNA between tumor and normal tissues in twenty cancer types based on the TCGA database. (**B**) The expression level of *KLRB1* mRNA between tumor and normal tissues in twenty-seven cancer types based on the integrated database from TCGA and GTEx datasets. **(C-F)** Representative IHC images of CD161 expression in glioma, lung cancer, brain cortex, and normal lung tissues.

Consistent high expression level of *KLRB1* mRNA were observed in tumor samples of BRCA, CHOL, COAD, ESCA, GBM, HNSC, KIRH, KIRC, LGG, LIHC, LUAD, LUSC, STAD, and THCA than normal tissue based on both comparisons (Figure 1A). Considering that the number size of normal tissue in the TCGA database is too small to be statistically convincing (e.g., TCGA glioblastoma multiforme (GBM) cohort has only five normal controls), we integrated the GTEx and TCGA databases to reflect the *KLRB1* mRNA expression landscape in a more convincing manner, and found that *KLRB1* mRNA level was consistently elevated in most cancers including ACC, BLCA, CESC, CHOL, COAD, ESCA, GBM, HNSC, KIRC, LAML, LGG, LUSC, OV, PAAD, PRAD, SKCM, STAD, TGCT, THCA, and UCS compared to GTEx normal controls (Figure 1B). To detect the CD161 protein expression profiles in human tissues, we evaluated it in various tumor and normal tissues using the HPA database. As showed in immunohistochemistry results (Figure 1C & D), CD161 protein was mainly distributed in cytoplasm or membrane, and was upregulated in several cancers such as glioma and lung cancer compared with corresponding normal tissues. Detailed information of IHC results was summarized in Table 1.

### The Association between *KLRB1*/CD161 Expression and Cancer Patient's Prognosis

**Figure 2:**
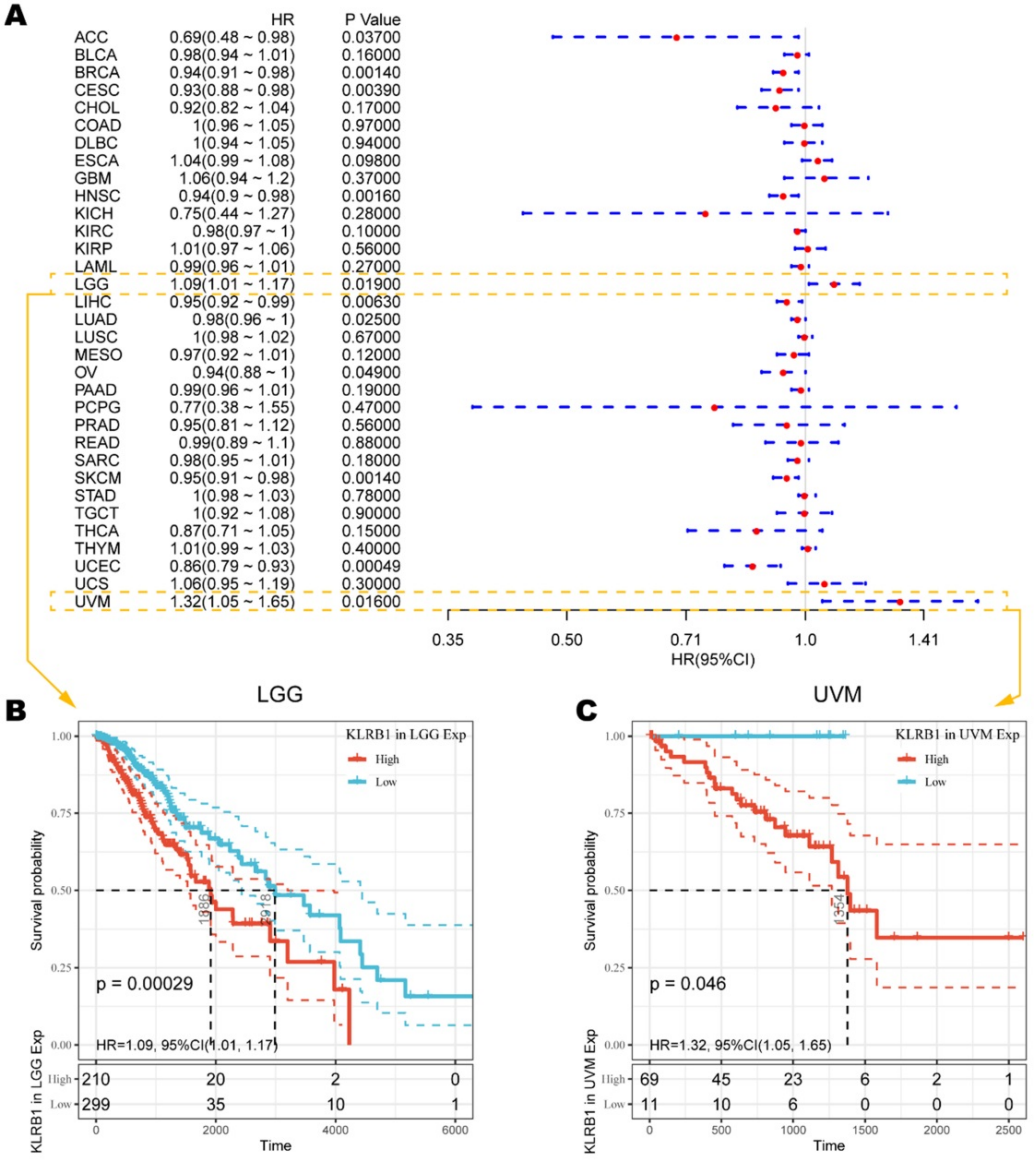
Survival analysis comparing the high and low expression of *KLRB1* on overall survival across different cancers in the TCGA dataset. **(A)** Forest plot displaying the impact of high expression of *KLRB1* mRNA on OS across thirty-three cancer types. **(B-C)** The correlation between high *KLRB1* mRNA expression and unfavorable prognosis in LGG and UVM cohorts.

To understand how CD161 impacts patient prognosis, we used cox regression model based on TCGA RNA-seq and clinical data to analyze the prognosis of thirty-three TCGA cancer types. As shown in Figure 2A, elevated *KLRB1* mRNA expression was significantly related to prolonged OS time in ACC (HR = 0.69, 95%CI = 0.48-0.98, *P* = 0.0370), BRCA (HR = 0.94, 95%CI = 0.91-0.98, *P* = 0.0014), CESC (HR = 0.93, 95%CI = 0.88-0.98, *P* = 0.0039), HNSC (HR = 0.94, 95%CI = 0.90-0.98, *P* = 0.0016), LIHC (HR = 0.95, 95%CI = 0.92-0.99, *P* = 0.0063), LUAD (HR = 0.98, 95%CI = 0.96-1.00, *P* = 0.0250), OV (HR = 0.94, 95%CI = 0.88-1.00, *P* = 0.0490), SKCM (HR = 0.95, 95%CI = 0.91-0.98, *P* = 0.0014), and UCEC (HR = 0.86, 95%CI = 0.79-0.93, *P* = 0.0005). On the contrary, increased *KLRB1* mRNA level was uniquely associated with unfavorable outcome in LGG (HR = 1.09, 95%CI = 1.01-1.17, *P* = 0.0190) and UVM (HR = 1.32, 95%CI = 1.05-1.65, *P* = 0.0160), the Kaplan-Meier survival curves of which were displayed as Figure 2B. And the survival curves with significance were summarized in Figure S1.

### High CD161 Expression Correlates with Immune Infiltration in LGG and UVM

**Figure 3:**
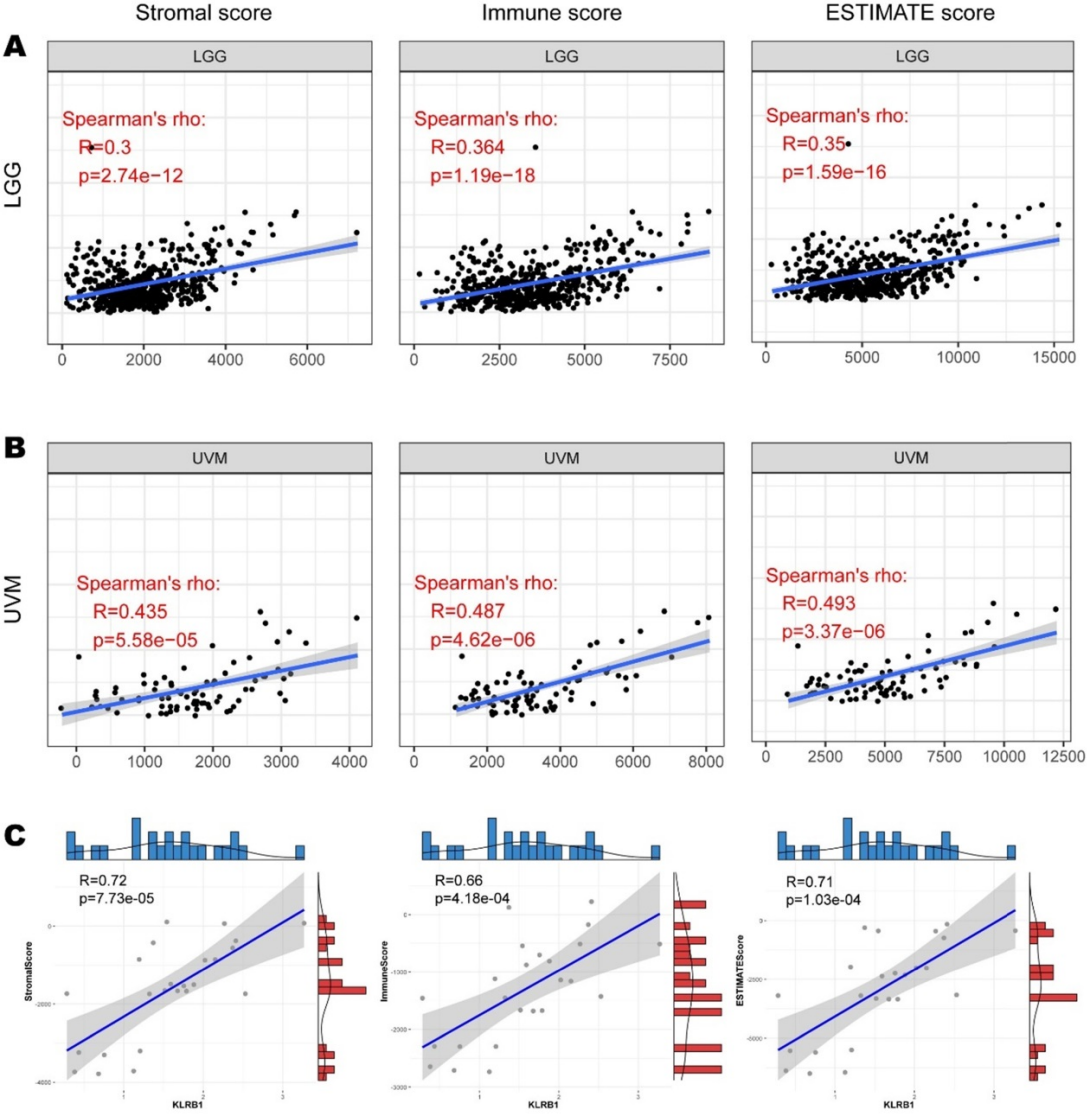
Correlation between CD161 expression with immune abundance based on ESTIMATE algorithm. (**A-B**) The correlations between CD161 with Stromal Score, Immune Score, and Estimate Score in LGG and UVM were displayed as scatter plots. (**C**) The correlations between CD161 with Stromal Score, Immune Score, and Estimate Score in 24 glioma samples.

To explore whether CD161 affects the prognosis of patients with LGG or UVM via interventions in immune infiltration, we investigated the relationship between CD161 and tumor purity based on the ESTIMATE algorithm. CD161 expression was positively correlated with Stromal Score, Immune Score, and ESTIMATE Score in both LGG and UVM cohorts (Figure 3A & B). And the correlations between CD161 and those scores across TCGA cancer types were displayed in Figure S2-4. We next used our samples to investigate whether CD161 correlates with tumor purity within glioma. In consistent with the TCGA cohort, CD161 expression was significantly correlated to tumor purity, as it was positively associated with stromal, immune and ESTIMATE scores (Figure 3C).

**Figure 4:**
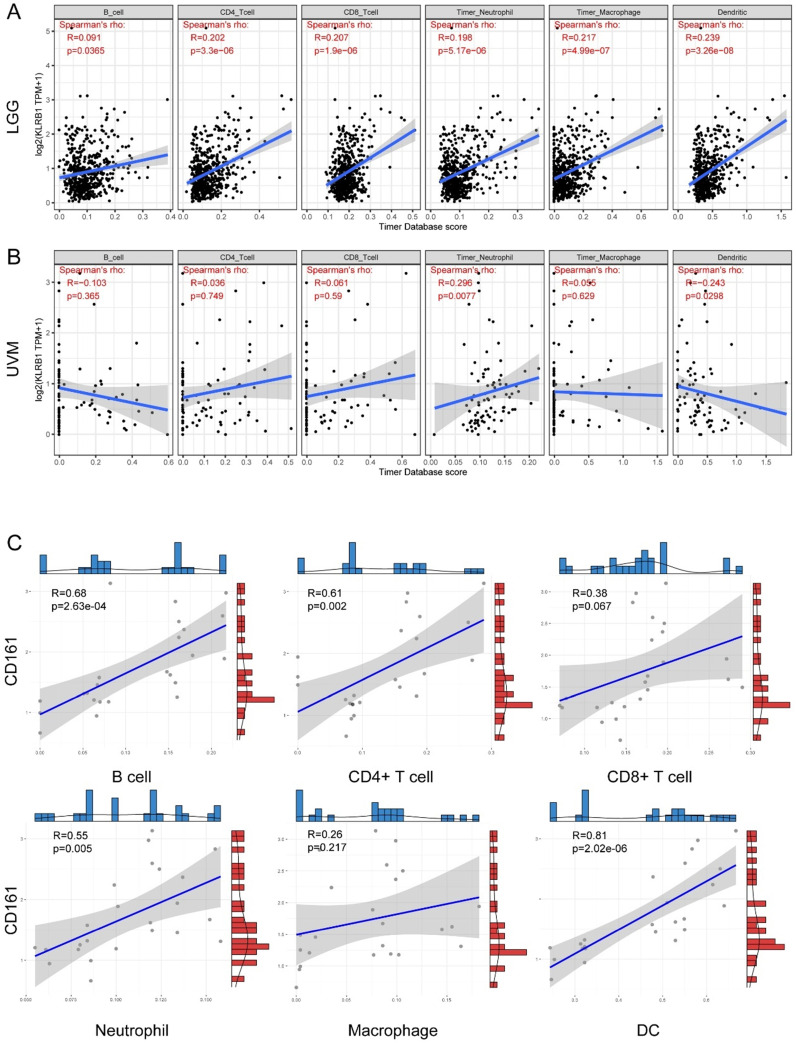
Correlation between CD161 expression with six immune infiltration levels based on TIMER algorithm. (**A-B**) The correlations in LGG and UVM were displayed as scatter plots. (**C**) The correlations in 24 glioma samples.

Regarding specific immune infiltrates. In general, CD161 expression was significantly and positively correlated with abundance of B cells, CD4+ T cells, CD8+ T cells, neutrophils, macrophages, and dendritic cells in LGG (Figure 4A). But positive correlations were only identified between CD161 expression and neutrophil infiltration in UVM (Figure 4B). In our samples, positive correlations were observed between CD161 expression and B cells, CD4+ T cells, neutrophils, and dendritic cells (Figure 4C), which was similar to the results obtained from the TCGA cohort.

### Relationship between CD161 Expression with Immune Checkpoints, TMB, and MSI

**Figure 5:**
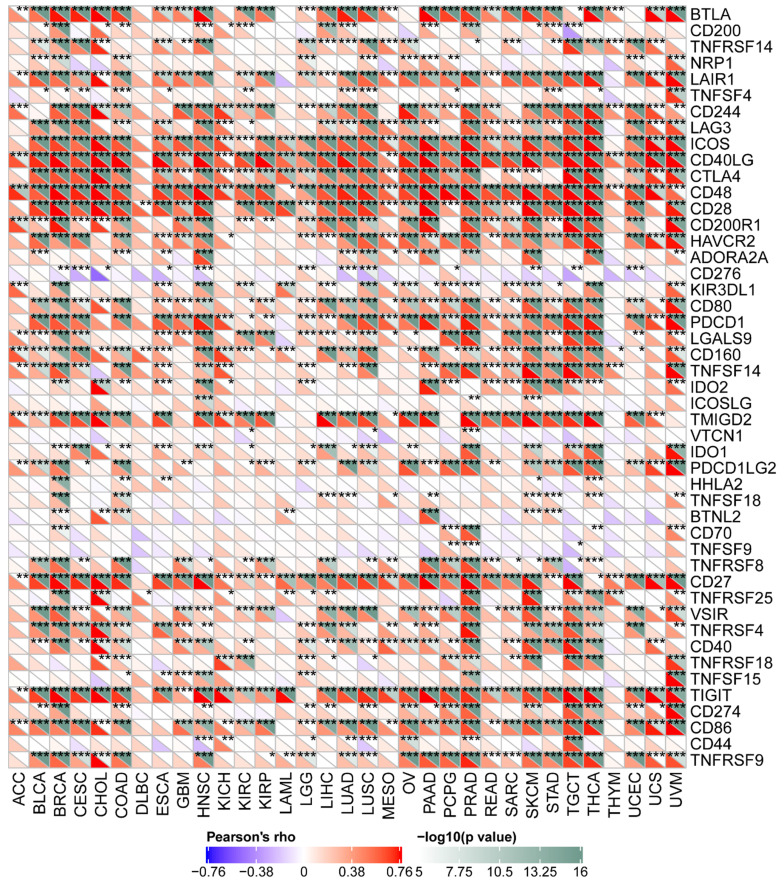
Correlation between CD161 expression with expressions of immune checkpoint genes across thirty-three cancer types (***P < 0.001).

The pan-cancer correlations between CD161 and immune checkpoints were displayed in Figure 5. In most cancers, except ACC, DLBC, and THYM, robust and significant relationships existed between CD161 and recognized immune checkpoints including B- and T-lymphocyte attenuator (BTLA), CD244, inducible T cell costimulator (ICOS), CD40 ligand, CD48, CD28, CD200 receptor 1, transmembrane and immunoglobulin domain containing 2 (TMIGD2), CD27, TIGIT, and CD86. This suggested a potential synergy of CD161 with known immune checkpoints.

**Figure 6:**
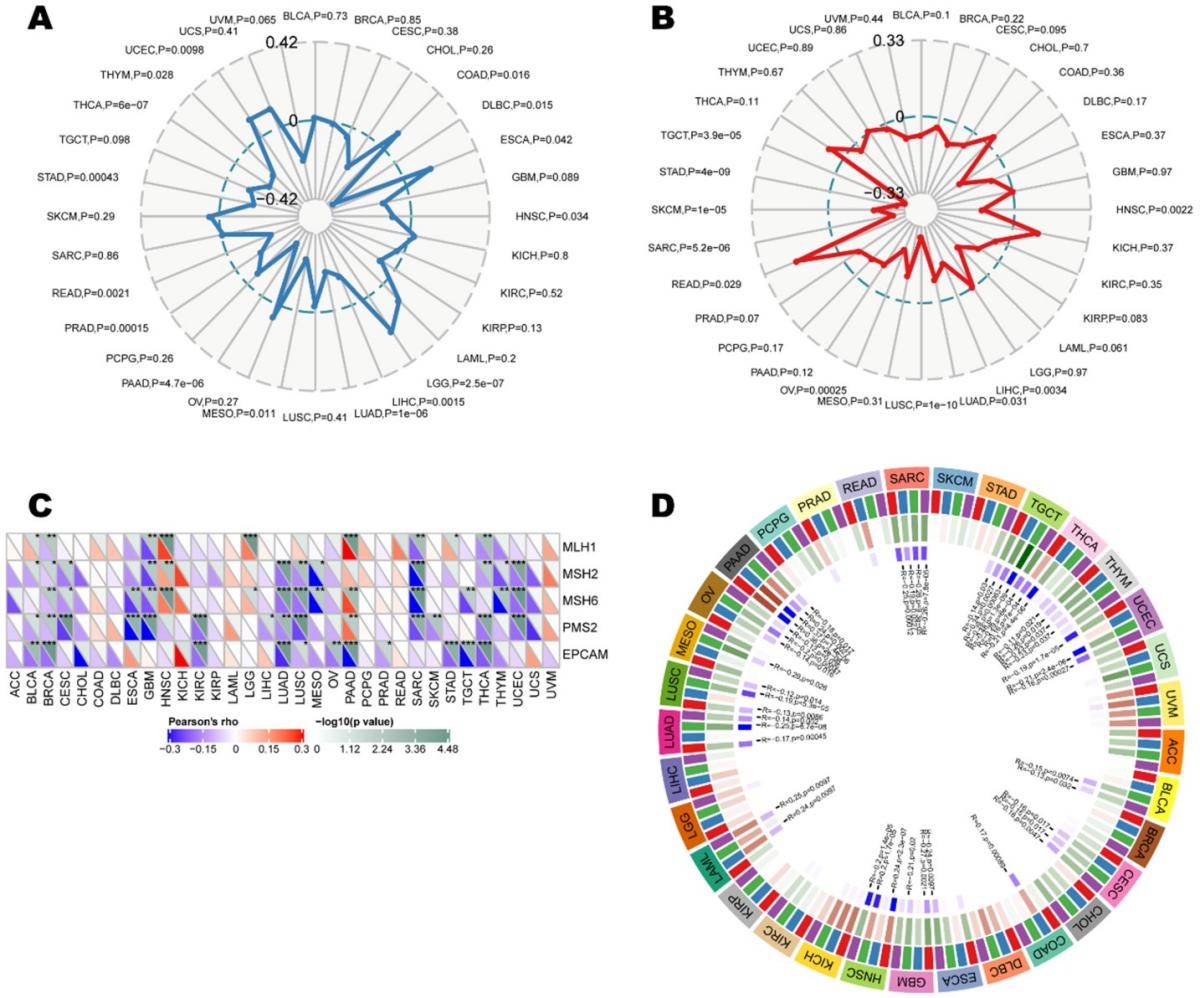
Correlations between CD161 expression with ICB efficacy predictors, including (**A**) tumor mutation burden, (**B**) microsatellite instability, (**C**) mismatch repair genes, as well as (**D**) DNA methyltransferases across thirty-three cancer types.

As indicators of a series of genomes or transcriptomes related to cancer initiation and development, tumor mutation burden, microsatellite instability, mismatch repair, and methylation were independent predictors of ICB efficacy. We herein examine the relationship between CD161 and those indicators to investigate whether CD161 affects tumorigenesis by participating in the process of genetic and/or transcriptional alterations. CD161 expression was negatively correlated with TMB in LUAD, PAAD, PRAD, STAD, and THCA (*P* < 0.001), while it was solely and positively correlated with TMB in LGG (*P* < 0.001) (Figure 6A). Moreover, MSI-low tumors expressed higher level of CD161 than genetically stable ones in LUSC, OV, SARC, SKCM, STAD and TGCT cohorts (*P* < 0.001, Figure 6B). In addition, we examined the correlation between CD161 expression and several essential MMR signatures. CD161 expression was positively correlated with MutL homolog 1 (MLH1), MutS homolog 2 (MSH2) and MutS homolog 6 (MSH6) in HNSC, LGG, and PAAD. In contrast, it was negatively correlated with these molecules in BLCA, BRCA, ESCA, GBM, LUAD, LUSC, SARC, THCA, and UCEC (Figure 6C). In terms of the relationship between CD161 expression and DNA methyltransferases, negative correlations were identified in BLCA, CESC, COAD, ESCA, GBM, HNSC, LAML, LUAD, LUSC, MESO, OV, PAAD, SARC, TGCT, THCA, THYM, and UCEC (Figure 6D). Despite the significances of these correlations, they were not such strong across all cancers with coefficients less than 0.6.

### CD161 impacts Patient Prognosis via intervening in Tumor Immunity

**Figure 7:**
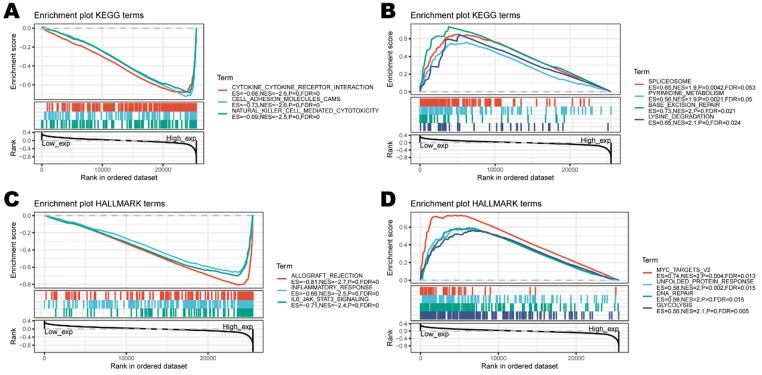
Functional enrichment of KEGG and HALLMARK terms on CD161 through GSEA. (**A-B**) The top three negative and positive enriched KEGG terms. (**C-D**) The top three negative and positive enriched HALLMARK terms.

To investigate the biological characteristics associated with CD161 in LGG and UVM, where CD161 was a significant risk factor. We performed gene set enrichment analysis (GSEA) and found that CD161 was mostly associated with immune responses (Figure 7C). Specifically, enriched KEGG pathways included 'cytokine-cytokine receptor interaction' and 'NK cell mediated cytotoxicity'. Meanwhile, HALLMARK enrichment analysis showed similar results that CD161 was involved in several terms such as 'inflammatory response' and 'IL6-JAK-STAT3 signaling'. This indicates that CD161 is involved in the regulation process within immune responses across human cancers, highly suggesting that CD161 influences patient prognosis by intervening in the immuno-oncological processes.

To clarify the specific cell types modulated by CD161 in tumor microenvironment, we summarized the correlations between CD161 expression and immune infiltrating levels in LGG and UVM based on sets of immunological markers using the TIMER2.0 database. We adjusted these results based on tumor purity, revealing strong and significant correlations between CD161 with CD8+ T cells, DCs, M2 macrophages, and Tex markers in both LGG and UVM cohorts (Table 2; Pearson's rho > 0.6, *P* < 0.001). Further re-examination using the GEPIA2 database revealed consistent results (Table 3).

## Discussion

In this report, we assessed the expression of CD161 in 33 different cancer types, revealing clear differences of pan-cancer CD161 expression between tumor and normal tissues. Analysis based on TCGA and GTEx data showed that CD161 expression was increased in most cancers including ACC, BLCA, CESC, CHOL, COAD, ESCA, GBM, HNSC, KIRC, LAML, LGG, LUSC, OV, PAAD, PRAD, SKCM, STAD, TGCT, THCA, and UCS compared with adjacent normal controls. Mainly expressed on NK cells, CD161 expression level may reflect the abundance of these two immune infiltrates in tumor microenvironment indirectly. And we suppose that CD161 plays a delicate role in tumorigenesis based on the differential expression profiles.

Although new therapies, such as targeted therapy and immunotherapy, have encouraging great success, current management of cancer like glioma cannot reach a favorable remission 29. Several identified genetic and/or transcriptional indicators including *IDH* mutation, O-6-methylguanine-DNA methyltransferase (MGMT) promoter methylation, and 1p/19q co-deletion were found to be closely related to glioma prognosis 30. Much like those indicators, our analyses demonstrated the potential of CD161 in risk stratification and prognostic prediction. And similar to PD-1/PD-L1 axis, CD161/CLEC2D pathway was suggested to mediate immune response in the oncologic context. Moreover, the significant correlation between elevated CD161 expression and worse prognosis in patients with LGG or UVM is similar to that of CD96 31, another promising immune checkpoint.

Another key finding of this study is that the CD161 expression is highly associated with immune infiltration. CD161 expression is positively correlated with the abundance of immune infiltrates, especially CD8+ T cells, DCs, macrophages, Tregs and Tfh in various cancers. These results were validated using our own samples obtained from Xiangya hospital. But further work will be necessary to establish whether CD161 exerts such functions. CD161 can affect patient prognosis by influencing immune processes, as CD161 was involved in the several immune terms (KEGG and HALLMARK) in LGG and UVM. Notably, CD161 was positively associated with markers of general T cell, DC, and exhausted T cell, suggesting CD161 impacted patient survival in an immunity-depended manner. Although the distinct infiltration landscape in different tumors may influence our results, it is reasonable to speculate that CD161 can influence the path of immune infiltrates in tumor microenvironment, and may alter the distribution and subsequent interplays with malignancies, leading to different survival outcomes in certain cancers.

The study has some limitations. First, there is no experimental validation of the predicted outcomes. Further studies should conduct the experimental validation by different methods, e.g., fluorescence quantitative polymerase chain reaction (qPCR), western blotting and immunocytochemistry. In addition, for diseases with long-term evolution, especially cancers. At least in some stages, transcriptomic profiles do not necessarily reflect the proteomics. Although we compared the differences in CD161 expression between cancer and normal tissues at the mRNA and protein levels, it remains debatable whether the differences in the proteins are necessarily associated with the malignancy of cancers. Moreover, although we conducted validation using our own samples, the correlations obtained in this study may not be applicable in other study cohorts due to the high heterogeneity across different populations. Therefore, experimental and clinical validations of the predicted results are required.

In conclusion, we applied an integrated bioinformatics approach, indicating that CD161 could mediate immune infiltration and influence patient prognosis in pan-cancer. Our findings demonstrate that CD161 has potential as a prognostic biomarker and provide a new orientation for the treatment of these malignancies. We believe that immunotherapy combining CD161 blockade and existing checkpoint inhibitors may be a highly effective and feasible approach against these unpleasing tumors, especially brain gliomas for which CD161 is a unique risk factor.

## Supplementary Material

Supplementary figures and table.

## Figures and Tables

**Table 1:** Clinical information and relative scores of immunohistochemistry results

Protein	Tissue	Histological type	Age	Gender	Location	Quantity	Intensity	Relative score
CD161	Glioma	Malignant glioma (High grade)	47	Male	C/M^1^	> 75%	Moderate	8
CD161	Lung cancer	Squamous cell carcinoma	65	Male	C/M^1^	> 75%	Moderate	8
CD161	Cerebral cortex	Glial cells	64	Female	C/M^1^	< 25%	Weak	1
CD161	Lung	Alveolar cells	67	Female	C/M^1^	< 25%	Moderate	2

^1^ C/M: Cytoplasmic/membranous.

**Table 2:** Correlations between CD161 and immune cell markers in TIMER2.0 (***P < 0.001)

Description	Markers	LGG (n = 516)				UVM (n = 80)			
		None		Purity		None		Purity	
		rho	*P*	rho	*P*	rho	*P*	rho	*P*
CD8+ T cell	CD8A	0.558	***	0.536	***	0.693	***	0.680	***
	CD8B	0.491	***	0.448	***	0.719	***	0.716	***
T cell (general)	CD3D	0.741	***	0.719	***	0.685	***	0.670	***
	CD3E	0.814	***	0.802	***	0.727	***	0.716	***
	CD2	0.806	***	0.792	***	0.718	***	0.708	***
B cell	CD19	0.356	***	0.310	***	0.385	***	0.360	***
	CD79A	0.322	***	0.309	***	0.329	***	0.320	***
Monocyte	CD86	0.425	***	0.397	***	0.747	***	0.737	***
	CD115 (CSF1R)	0.264	***	0.211	***	0.543	***	0.529	***
TAM	CCL2	0.396	***	0.373	***	0.611	***	0.598	***
	CD68	0.453	***	0.432	***	0.285	***	0.266	***
	IL10	0.425	***	0.412	***	0.583	***	0.564	***
M1 Macrophage	INOS (NOS2)	-0.092	0.037	-0.093	0.042	0.167	0.138	0.146	0.205
	IRF5	0.371	***	0.335	***	0.595	***	0.575	***
	COX2 (PTGS2)	0.211	***	0.181	***	0.350	***	0.332	***
M2 Macrophage	CD163	0.450	***	0.450	***	0.668	***	0.654	***
	VSIG4	0.274	***	0.238	***	0.613	***	0.599	***
	MS4A4A	0.452	***	0.452	***	0.724	***	0.712	***
Neutrophils	CD66b (CEACAM8)	-0.011	0.801	-0.016	0.723	NA	NA	NA	NA
	CD11b (ITGAM)	0.349	***	0.311	***	0.471	***	0.487	***
	CCR7	0.552	***	0.534	***	0.769	***	0.762	***
NK cell	KIR2DL1	0.159	***	0.169	***	0.349	***	0.368	***
	KIR2DL3	0.308	***	0.313	***	0.450	***	0.421	***
	KIR2DL4	0.377	***	0.382	***	0.509	***	0.484	***
	KIR3DL1	0.235	***	0.231	***	0.420	***	0.391	***
	KIR3DL2	0.209	***	0.238	***	0.603	***	0.585	***
	KIR3DL3	-0.009	0.836	0.000	0.998	0.373	***	0.334	***
	KIR2DS4	0.343	***	0.333	***	0.300	***	0.266	***
Dendritic cell	HLA-DPB1	0.625	***	0.615	***	0.661	***	0.648	***
	HLA-DQB1	0.527	***	0.520	***	0.605	***	0.584	***
	HLA-DRA	0.613	***	0.604	***	0.713	***	0.700	***
	HLA-DPA1	0.620	***	0.614	***	0.657	***	0.641	***
	BCDA-1 (CD1C)	0.440	***	0.416	***	0.554	***	0.541	***
	BDCA-4 (NRP1)	0.378	***	0.405	***	0.547	***	0.524	***
	CD11c (ITGAX)	0.304	***	0.268	***	0.549	***	0.530	***
Th1	TBX21	0.519	***	0.524	***	0.706	***	0.696	***
	STAT4	0.146	***	0.116	***	0.488	***	0.479	***
	STAT1	0.393	***	0.394	***	0.505	***	0.482	***
	IFN-γ (IFNG)	0.337	***	0.310	***	0.514	***	0.503	***
	TNF-α (TNF)	0.150	***	0.119	***	0.619	***	0.608	***
Th2	GATA3	0.450	***	0.413	***	0.625	***	0.618	***
	STAT6	0.377	***	0.343	***	0.191	0.089	0.162	0.158
	STAT5A	0.354	***	0.310	***	0.061	0.588	0.083	0.471
	IL13	-0.092	0.037	-0.075	0.100	0.086	0.447	0.034	0.768
Tfh	BCL6	-0.165	***	-0.141	***	0.213	0.058	0.234	***
	IL21	0.078	0.078	0.075	0.101	0.322	***	0.274	***
Th17	STAT3	0.280	***	0.283	***	0.282	***	0.253	***
	IL17A	0.065	0.138	0.057	0.214	NA	NA	NA	NA
Treg	FOXP3	0.009	0.831	0.014	0.758	0.458	***	0.430	***
	CCR8	0.259	***	0.274	***	0.428	***	0.394	***
	STAT5B	-0.177	***	-0.111	***	0.266	***	0.251	***
	TGFβ (TGFB1)	0.285	***	0.242	***	0.440	***	0.419	***
Tex	PD-1 (PDCD1)	0.528	***	0.512	***	0.666	***	0.660	***
	CTLA4	0.467	***	0.432	***	0.555	***	0.534	***
	LAG3	0.282	***	0.304	***	0.593	***	0.577	***
	TIM-3 (HAVCR2)	0.421	***	0.396	***	0.734	***	0.723	***
	GZMB	0.516	***	0.523	***	0.652	***	0.643	***
FDC	BAFF (TNFSF13B)	0.312	***	0.014	0.758	0.642	***	0.430	***
	CD35 (CR1)	0.330	***	0.287	***	0.689	***	0.680	***
	CD21 (CR2)	0.068	0.125	0.010	0.829	0.272	0.015	0.246	0.031
	CD44	0.382	***	0.353	***	-0.003	0.976	0.032	0.886
	CD29 (ITGB1)	0.373	***	0.379	***	0.117	0.303	0.111	0.337

**Table 3:** Correlations between CD161 and markers of CD8+ T cell, general T cell, M2 Macrophage, DC, Th1, and Treg in GEPIA2 (***P < 0.001)

Description	Markers	LGG		UVM	
		Tumor		Tumor	
		R	*P*	R	*P*
CD8+ T cell	CD8A	0.56	***	0.72	***
	CD8B	0.51	***	0.74	***
T cell (general)	CD3D	0.75	***	0.72	***
	CD3E	0.84	***	0.75	***
	CD2	0.81	***	0.75	***
M2 Macrophage	CD163	0.49	***	0.74	***
	VSIG4	0.29	***	0.62	***
	MS4A4A	0.46	***	0.73	***
DC	HLA-DPB1	0.62	***	0.67	***
	HLA-DRA	0.61	***	0.75	***
	HLA-DPA1	0.61	***	0.68	***
Th1	TBX21	0.54	***	0.73	***
	TNF-α (TNF)	0.16	***	0.60	***
Treg	PD-1 (PDCD1)	0.54	***	0.67	***
	CTLA4	0.47	***	0.55	***
	LAG3	0.27	***	0.62	***
	TIM-3 (HAVCR2)	0.42	***	0.77	***
	GZMB	0.55	***	0.69	***

## Abbreviations

NK cell: natural killer cell
OS: overall survival
DC: dendric cell
Tex: exhausted T cell
CTLA-4: cytotoxic T lymphocyte associated protein 4
PD-1: programmed death-1
ICB: immune checkpoint blockade
KLRB1: killer cell lectin-like receptor subfamily B member 1
RNA-seq: RNA sequencing
NSCLC: non-small cell lung cancer
TCGA: The Cancer Genome Atlas
FPKM: fragments per kilobase million
TPM: transcripts per kilobase million
GTEx: genotype-tissue expression
HPA: Human Protein Atlas
HR: hazard ratio
95%CI: 95% confidence interval
TMB: tumor mutation burden
MSI: microsatellite instability
MMR: mismatch repair
TIMER: Tumor IMmune Estimation Resource
TAM: tumor-associated macrophage
Th: helper T cell
GEPIA: Gene Expression Profiling Interactive Analysis
ANOVA: analysis of variance
BTLA: B- and T-lymphocyte attenuator
ICOS: inducible T cell costimulator
TMIGD2: transmembrane and immunoglobulin domain containing 2
MLH: MutL homolog
MSH: MutS homolog
GSEA: gene set enrichment analysis
MGMT: O-6-methylguanine-DNA methyltransferase
qPCR: quantitative polymerase chain reaction
ACC: adrenocortical carcinoma
BLCA: bladder urothelial carcinoma
BRCA: breast invasive carcinoma
CESC: cervical squamous cell carcinoma
CHOL: cholangiocarcinoma
COAD: colon adenocarcinoma
DLBC: diffuse large B-cell lymphoma
ESCA: esophageal carcinoma
GBM: glioblastoma multiforme
HNSC: head and neck squamous cell carcinoma
KIRC: kidney renal clear cell carcinoma
KIRP: kidney renal papillary cell carcinoma
LAML: acute myeloid leukemia
LGG: brain lower grade glioma
LIHC: liver hepatocellular carcinoma
LUSC: lung squamous cell carcinoma
MESO: mesothelioma
OV: ovarian serous cystadenocarcinoma
PAAD: pancreatic adenocarcinoma
PCPG: pheochromocytoma and paraganglioma
PRAD: prostate adenocarcinoma
READ: rectum adenocarcinoma
SARC: sarcoma
SKCM: skin cutaneous melanoma
STAD: stomach adenocarcinoma
TGCT: testicular germ cell tumor
THCA: thyroid carcinoma
THYM: thymoma
UCEC: uterine corpus endometrial carcinoma
UCS: uterine carcinosarcoma
UVM: uveal melanoma
