# Supplementary material for: CD161, a promising Immune Checkpoint, correlates with Patient Prognosis: A Pan-cancer Analysis
Source: J Cancer. 2021 Sep 9;12(21):6588–99. doi: 10.7150/jca.63236 (PMC8489134; doi:10.7150/jca.63236)
Supplement: Supplementary file 1 — Supplementary figures and table. [file jcav12p6588s1.pdf]

Table S1. Clinical information of twenty-four samples in our study.

ID	Gender	Age	Histological type	WHO Grade
1	male	45	Glioblastoma	IV
2	male	45	Glioblastoma	IV
3	male	27	Astrocytoma	II
4	male	59	Oligodendroglioma	II
5	male	27	Astrocytoma	II
6	male	27	Astrocytoma	II
7	male	75	Glioblastoma	IV
8	male	60	Diffuse astrocytoma	II
9	male	22	Glioblastoma	IV
10	male	75	Glioblastoma	IV
11	male	22	Glioblastoma	IV
12	male	75	Glioblastoma	IV
13	female	54	Aplastic astrocytoma	III
14	male	60	Diffuse astrocytoma	II
15	female	49	Glioblastoma	IV
16	female	49	Glioblastoma	IV
17	male	59	Oligodendroglioma	II
18	male	60	Diffuse astrocytoma	II
19	male	45	Glioblastoma	IV
20	female	54	Aplastic astrocytoma	III
21	female	54	Aplastic astrocytoma	III
22	male	59	Oligodendroglioma	II
23	female	49	Glioblastoma	IV
24	male	22	Glioblastoma	IV

Figure S1

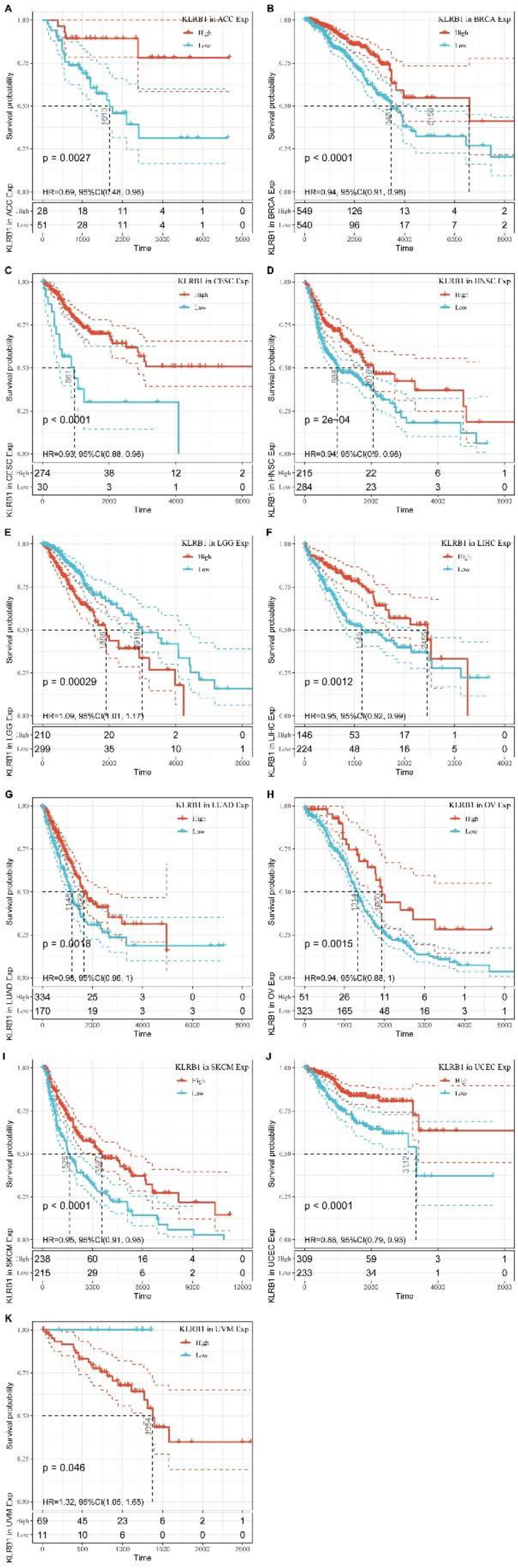
A
KLRB1 in ACC Exp
p = 0.0027
HR=0.69, 95%CI(0.48, 0.98)
B
KLRB1 in BRCA Exp
p < 0.0001
HR=0.94, 95%CI(0.91, 0.98)
C
KLRB1 in CESC Exp
p < 0.0001
HR=0.93, 95%CI(0.88, 0.98)
D
KLRB1 in HNSC Exp
p = 2e-04
E
KLRB1 in LGG Exp
p = 0.00029
HR=1.09, 95%CI(1.01, 1.17)
F
KLRB1 in LIHC Exp
p = 0.0012
HR=0.95, 95%CI(0.92, 0.99)
G
KLRB1 in LUAD Exp
p = 0.0018
H
KLRB1 in OV Exp
p = 0.0015
HR=0.94, 95%CI(0.88, 1)
I
KLRB1 in SKCM Exp
p < 0.0001
HR=0.95, 95%CI(0.91, 0.98)
J
KLRB1 in UCEC Exp
p < 0.0001
HR=0.86, 95%CI(0.79, 0.93)
K
KLRB1 in UVM Exp
p = 0.046
HR=1.32, 95%CI(1.05, 1.65)
Survival probability
Time
High
Low

Figure S2

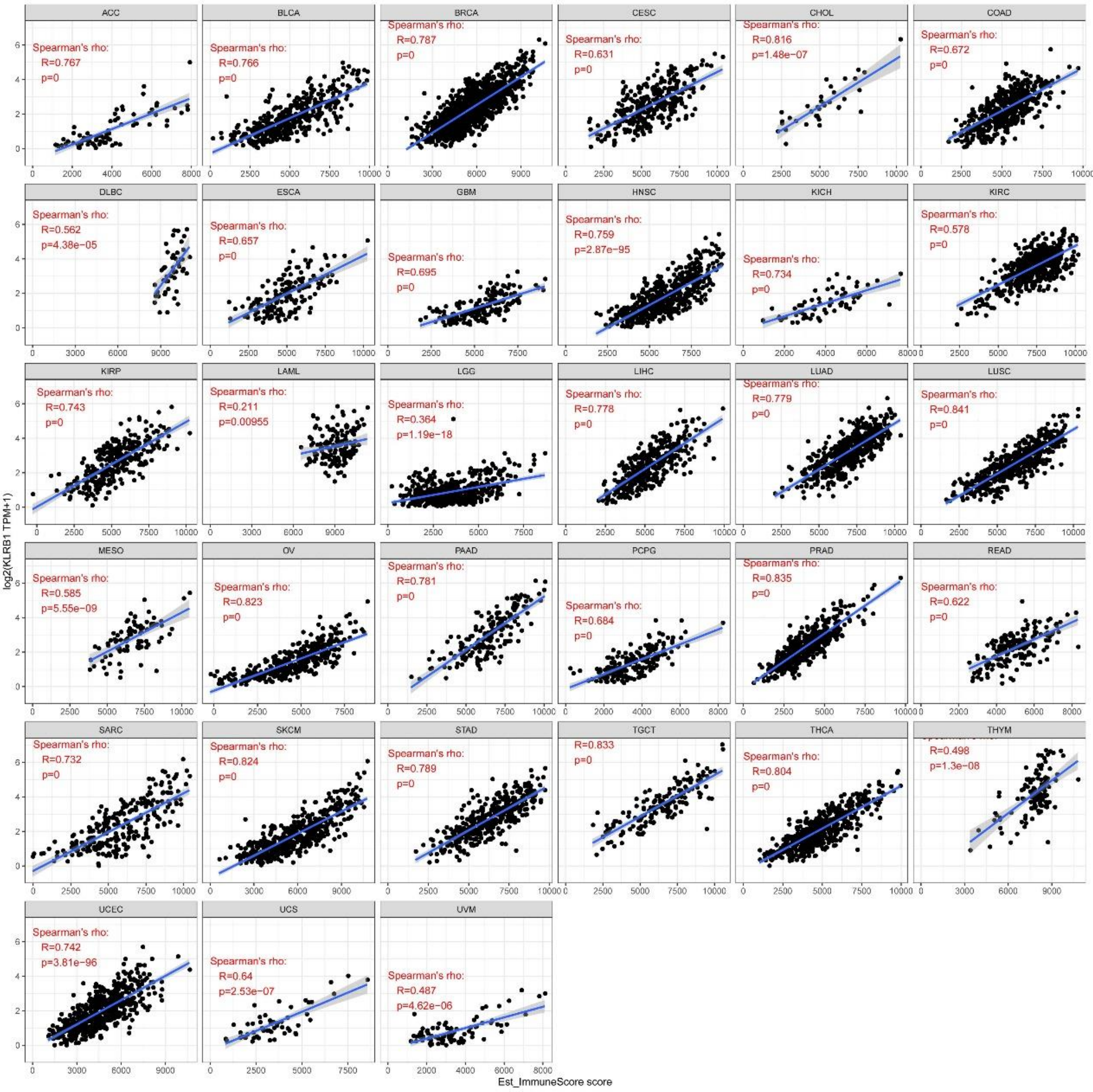

Figure S3

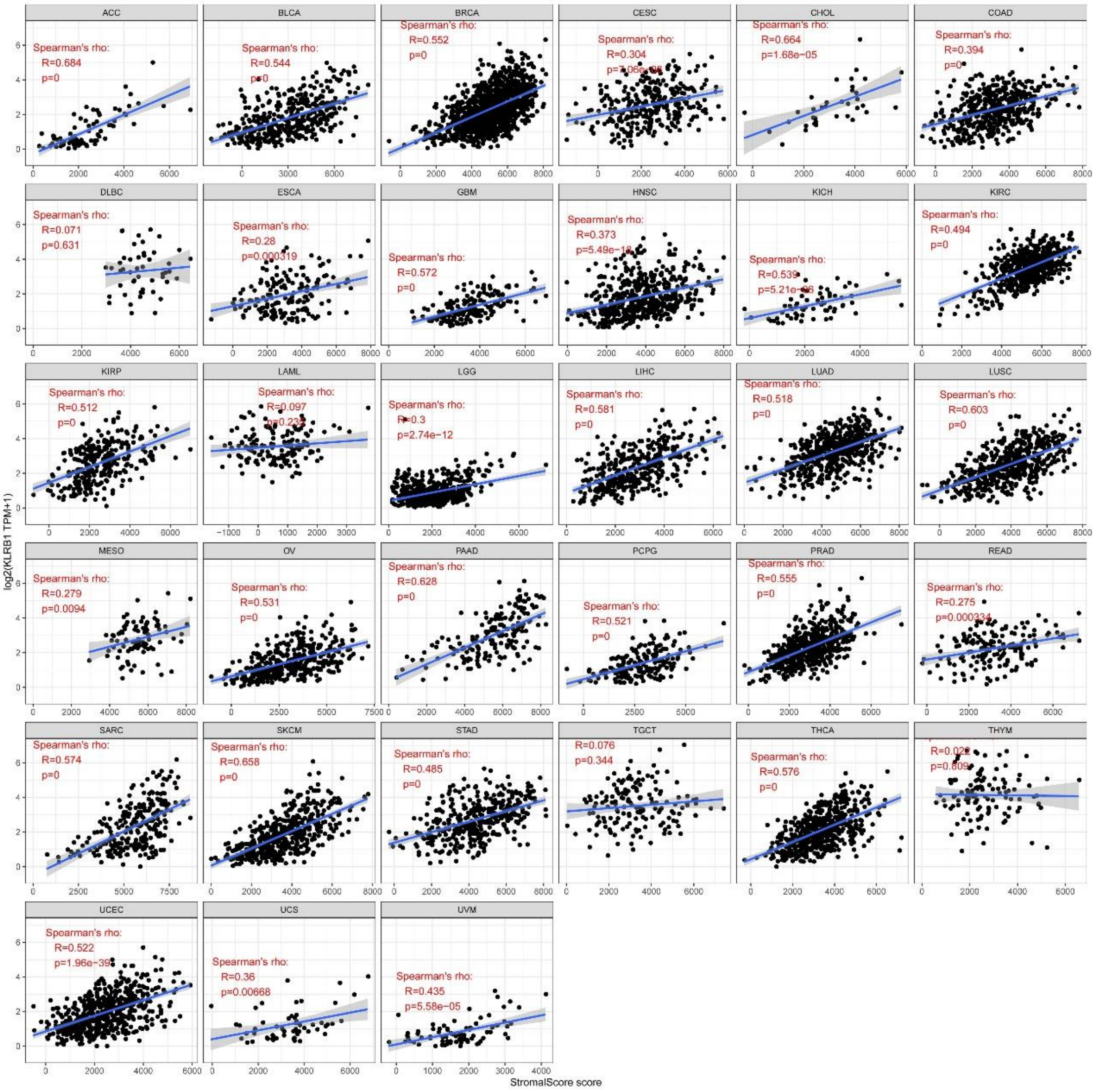

ACC
Spearman's rho:
R=0.684
p=0
BLCA
Spearman's rho:
R=0.544
BRCA
Spearman's rho:
R=0.552
p=0
CESC
Spearman's rho:
R=0.304
CHOL
Spearman's rho:
R=0.664
p=1.68e−05
COAD
Spearman's rho:
R=0.394
DLBC
Spearman's rho:
R=0.071
p=0.631
ESCA
Spearman's rho:
R=0.28
GBM
Spearman's rho:
R=0.572
p=0
HNSC
Spearman's rho:
R=0.373
KICH
Spearman's rho:
R=0.539
KIRC
Spearman's rho:
R=0.494
p=0
KIRP
Spearman's rho:
R=0.512
p=0
LAML
Spearman's rho:
LGG
Spearman's rho:
p=2.74e−12
LIHC
Spearman's rho:
R=0.581
p=0
LUAD
Spearman's rho:
R=0.518
p=0
LUSC
Spearman's rho:
R=0.603
p=0
MESO
Spearman's rho:
R=0.279
p=0.0094
OV
Spearman's rho:
R=0.531
p=0
PAAD
Spearman's rho:
R=0.628
p=0
PCPG
Spearman's rho:
R=0.521
p=0
PRAD
Spearman's rho:
R=0.555
p=0
READ
Spearman's rho:
R=0.275
SARC
Spearman's rho:
R=0.574
p=0
SKCM
Spearman's rho:
R=0.658
p=0
STAD
Spearman's rho:
R=0.485
p=0
TGCT
R=0.076
p=0.344
THCA
Spearman's rho:
R=0.576
p=0
THYM
UCEC
Spearman's rho:
R=0.522
UCS
Spearman's rho:
R=0.36
p=0.00668
UVM
Spearman's rho:
R=0.435
p=5.58e−05
log2(KLRB1 TPM+1)
StromalScore score

Figure S4

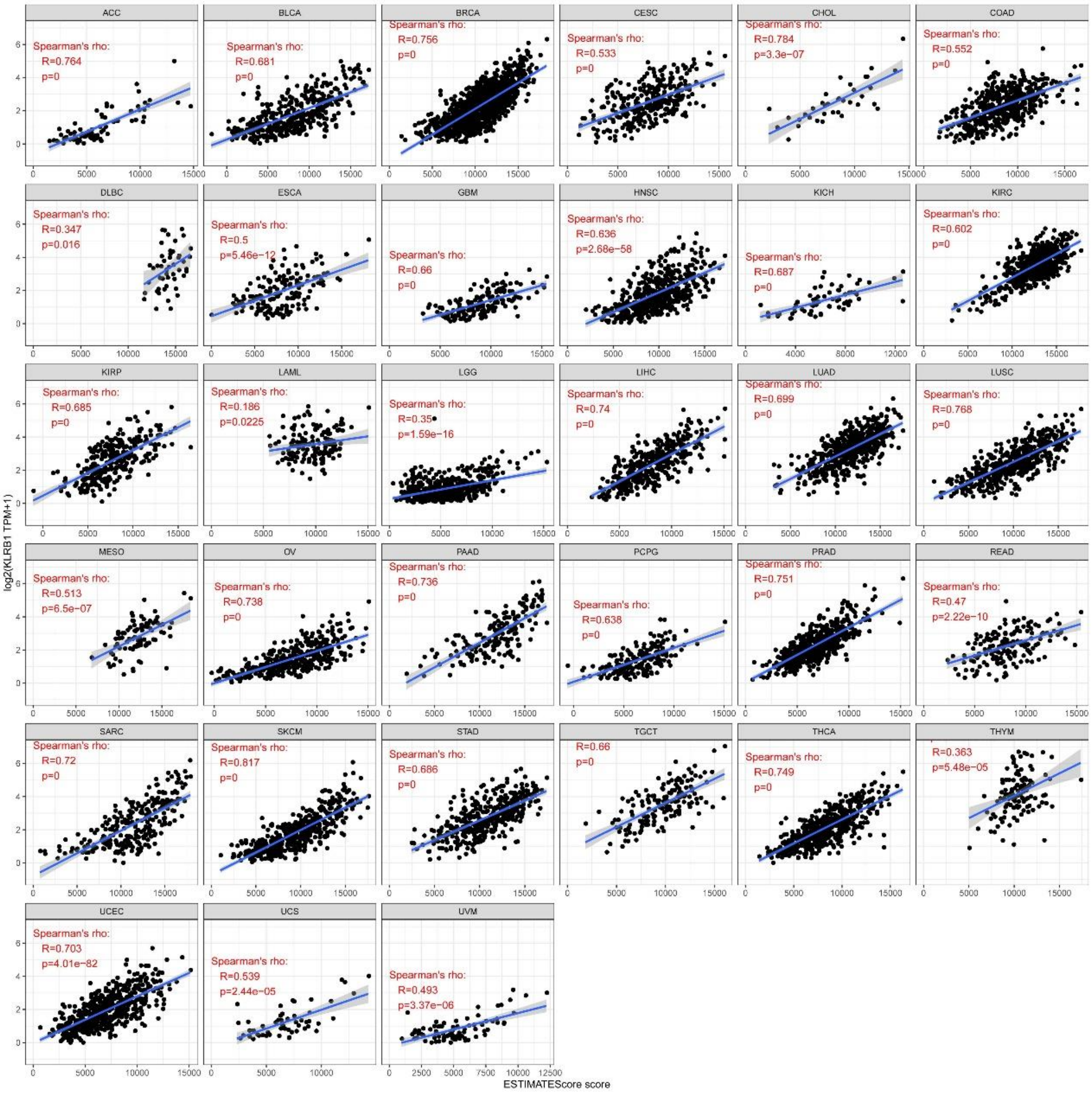

ACC
Spearman's rho:
R=0.764
p=0
BLCA
Spearman's rho:
R=0.681
p=0
BRCA
Spearman's rho:
R=0.756
p=0
CESC
Spearman's rho:
R=0.533
p=0
CHOL
Spearman's rho:
R=0.784
p=3.3e−07
COAD
Spearman's rho:
R=0.552
p=0
DLBC
Spearman's rho:
R=0.347
p=0.016
ESCA
Spearman's rho:
R=0.5
p=5.46e−12
GBM
Spearman's rho:
R=0.66
p=0
HNSC
Spearman's rho:
R=0.636
p=2.68e−58
KICH
Spearman's rho:
R=0.687
p=0
KIRC
Spearman's rho:
R=0.602
p=0
KIRP
Spearman's rho:
R=0.685
p=0
LAML
Spearman's rho:
R=0.186
p=0.0225
LGG
Spearman's rho:
R=0.35
p=1.59e−16
LIHC
Spearman's rho:
R=0.74
p=0
LUAD
Spearman's rho:
R=0.699
p=0
LUSC
Spearman's rho:
R=0.768
p=0
MESO
Spearman's rho:
R=0.513
p=6.5e−07
OV
Spearman's rho:
R=0.738
p=0
PAAD
Spearman's rho:
R=0.736
p=0
PCPG
Spearman's rho:
R=0.638
p=0
PRAD
Spearman's rho:
R=0.751
p=0
READ
Spearman's rho:
R=0.47
p=2.22e−10
SARC
Spearman's rho:
R=0.72
p=0
SKCM
Spearman's rho:
R=0.817
p=0
STAD
Spearman's rho:
R=0.686
p=0
TGCT
R=0.66
p=0
THCA
Spearman's rho:
R=0.749
p=0
THYM
R=0.363
p=5.48e−05
UCEC
Spearman's rho:
R=0.703
p=4.01e−82
UCS
Spearman's rho:
R=0.539
p=2.44e−05
UVM
Spearman's rho:
R=0.493
p=3.37e−06
log2(KLRB1 TPM+1)
ESTIMATEScore score
